# Low dietary vitamin E intake is associated with high risk of incident dementia among older adults: The Shanghai Aging Study

**DOI:** 10.3389/fnut.2022.1036795

**Published:** 2022-11-23

**Authors:** Su Liu, Jianfeng Luo, Zhenxu Xiao, Wanqing Wu, Xiaoniu Liang, Saineng Ding, Qianhua Zhao, Xianfeng Zhao, Yi Wang, Ding Ding

**Affiliations:** ^1^Department of Biostatistics, School of Public Health, Fudan University, Shanghai, China; ^2^NHC Key Laboratory of Health Technology Assessment, Fudan University, Shanghai, China; ^3^Institute of Neurology, Huashan Hospital, Fudan University, Shanghai, China; ^4^National Center for Neurological Disorders, Huashan Hospital, Fudan University, Shanghai, China; ^5^National Clinical Research Center for Aging and Medicine, Huashan Hospital, Fudan University, Shanghai, China; ^6^Danone Open Science Research Center for Life-Transforming Nutrition, Shanghai, China

**Keywords:** dementia, vitamin E, cognition, cohort study, risk

## Abstract

**Background:**

Growing evidence has shown the association between vitamin E intake and the risk of cognitive decline, but the conclusions were inconsistent. This study aimed to verify the hypothesis that vitamin E intake is associated with incident dementia and deterioration of global cognition.

**Materials and methods:**

We followed 1,550 non-demented community residents aged ≥60 years for an average of 5.2 years in the Shanghai Aging Study. Baseline vitamin E intake were measured by the Food Frequency Questionnaire. Cognitive function was evaluated by a battery of neuropsychological tests. Consensus diagnosis of incident dementia was made based on the DSM-IV criteria.

**Results:**

During the follow-up, 135 cases (8.7%) of incident dementia were identified. The incidence rates of dementia in low, low-medium, medium-high, and high vitamin E intake groups were 2.8, 1.5, 1.6, and 0.7 per 100 person-years, respectively (*P* < 0.001). Participants with low vitamin E intake had a significantly higher risk of incident dementia than those with higher intake [compared with the highest intake group: hazard ratio (HR) 2.34, 95% confidence interval (CI) 1.20–4.57] after adjusting for confounders. Vitamin E intake was negatively correlated to the rate of annual decline of Mini-Mental State Examination score with the adjustment of confounders (β = 0.019, *p* = 0.001).

**Conclusion:**

Vitamin E intake is negatively correlated with the risk of dementia in older adults. An appropriate high amount of vitamin E intake from the diet might be helpful to prevent future cognitive decline.

## Introduction

Dementia, a degenerative brain disease with symptoms, such as memory loss, language problems, and functional impairment, is a leading cause of disability in people older than 5 years (1). This disease is considered to start 20 years or more before the onset of symptoms, and the changes in the brain of patients in years are imperceptible (2–6). About 50 million people worldwide suffer from dementia, and this number is expected to increase to 152 million by 2050 (7). Disease burden of dementia is extremely high, especially in low-and middle-income countries (LMICs), where about two-thirds of people with dementia live (7). Dementia poses a huge challenge for policy makers, healthcare professionals, and family members (1).

Multiple mechanisms associated with disease onset and progression have been reported. One key mechanism implicated in dementia is oxidative stress, which may be modifiable through balanced diet and/or anti-oxidant supplements. The tocopherol and tocotrienol isoforms of vitamin E have multiple properties including potent anti-oxidant and anti-inflammatory characteristics, in addition to influences on immune function, cellular signaling and cholesterollowering (8),which offer a potentialtheoretical rationale for providing benefit for the treatment of Alzheimer’s disease (AD)-associated pathology (9, 10). Decreased concentrations of vitamin E in circulation have been found in individuals with AD. Vitamin E intake, particularly from dietary sources, may slow the progression of cognitive impairment (11). However, some studies indicate that vitamin E does not play a significant role in the prevention and treatment of AD (12, 13). And it may related to the different research methods and the dose of vitamin E intake. Therefore the conclusions about vitamin E and dementia were inconsistent. Currently, studies investigating the association between dietary vitamin E intake and the incidence of dementia mostly come from western populations. Prospective community-based studies are limited in Asian population to understand such an association.

The “Shanghai Aging Study” is a population-based cohort study conducted in China with the study design, operational procedures and diagnostic criteria similar to most cohort studies of memory in western countries (14). The current study intended to explore the relationship between dietary vitamin E intake and dementia risk by analyzing the data from baseline and the 5-year follow-up in the Shanghai Aging Study.

## Materials and methods

### Study participants

Participants aged 60 or older were recruited from an urban community of Shanghai, China in 2010–2011. We recruited participants who were permanent residents in the community, not living in nursing homes or other institutions, and could cooperate with the neuropsychological evaluation. Those suffering from mental retardation, schizophrenia and severe impairment in hearing, vision, or language were excluded. The current study included those dementia-free participants who provided the dietary data at baseline and completed the follow-up interview.

This study was approved by the Medical Ethics Committee of Huashan Hospital, Fudan University, Shanghai, China (No. 2009-195). All the participants and/or their legal guardians provided written informed consent to participate in the study.

### Measurement of consumption of nutrients

At baseline, daily dietary intake including frequency and amount over the past 12 months was measured for each participant using a 111-item interviewer-administered food frequency questionnaire (FFQ), which has been previously validated in Chinese population (15, 16). The FFQ include 81 groups of foods in 8 categories, such as staple food and animal meat. The FFQ also readjusted to suit the local diet in Shanghai, China, such as soy foods, fermented foods, salted foods, allium vegetables, and leafy vegetables. The recall period for FFQ was the past 12 months before the survey. Average daily intakes of dietary vitamin E, consumption of other major nutrients, and energy were calculated based on data from the FFQ and the “China Food Composition, 2nd Edition” (17).

### Neurological, neuropsychological assessments, and consensus diagnosis

Neurological examinations, Clinical Dementia Rating (CDR) (18), Lawton and Brody Activity of Daily Living (ADL) scale (19), neuropsychological assessments, and consensus diagnoses were conducted for all the participants both at baseline and the follow-up.

Neuropsychological tests covered the domains of global cognition, executive function, spatial construction, memory, language, and attention, including: (1) Mini-Mental State Examination (20); (2) Conflicting Instructions Task (Go/No Go Task) (21); (3) Stick Test (22); (4) Modified Common Objects Sorting Test (22); (5) Auditory Verbal Learning Test (23); (6) Modified Fuld Object Memory Evaluation (24); (7) Trail-making tests A and B (25); (8) RMB (Chinese currency) test (26). All the tests were administered by study psychometrists based on the education level of each participant: Tests (1) to (5)and (7) were used for participants with at least 6 years of education, while tests (1) to (4), (6) and (8) were used for those with less than 6 years of education (27). Normative data and a detailed description of these tests have been reported elsewhere (27, 28). All tests were conducted in Chinese within 90 min. Consensus diagnosis of dementia was reached based on the DSM-IV criteria by a professional group with the neurologist, neuropsychologist and neuroepidemiologist.

### Confounding factors

At baseline, we obtained a group of potential confounders related to diet and cognition, i.e., age, gender, education year, physical activity, cognitive activity, alcohol drinking, and cigarette smoking status were collected by an interviewer-administered questionnaire. Smoking or drinking was defined as whether the participant was a current smoker or drinker (14). Cognitive activity was assessed using Shanghai Cognitive Activities Scale. Physical activity was determined based on questionnaires and further transformed into metabolic equivalent values (29).

Body-mass index (BMI) was calculated by the body weight in kilograms divided by the squared height in meter. Depressive symptoms within the past week were assessed by the Center for Epidemiologic Studies Depression Scale (CES-D) (27). DNA was extracted from blood or saliva samples of participants. We used the Taqman SNP method (30) to conduct the APOE genotyping. APOE-ε4 positive was defined as the presence of at least one ε4 allele.

### Follow-up procedure

From 1 April 2014, to 31 December 2016, dementia-free participants at baseline were contacted and invited for follow-up interviews. A face-to-face neurological and neuropsychological assessment was conducted using the same procedure as the baseline. New-onset dementia cases were determined by the consensus diagnosis with the same method and criteria at the baseline.

### Statistical analysis

Continuous variables were expressed as the mean and standard deviation (SD), and categorical variables were expressed as number and frequencies (%). The Student’s *t*-test, Wilcoxon rank-sum test, Pearson Chi-square test, and Fisher’s exact test were used to compare continuous and categorical variables. Incidence rate of dementia was calculated as the number of new-onset cases divided by the total person-years of follow-up. The Kaplan–Meier curve was performed for the cumulative incidence rate of dementia by follow-up time.

The quartile of the vitamin E intake was used as the cut-off points to categorize the study participants into four groups of vitamin E intake: low intake group (<13.20 mg/d); low-medium intake group (13.20∼17.54 mg/d); medium-high intake group (17.54∼23.63 mg/d); and high intake group (>23.63 mg/d). Multivariate Cox regression model was used to estimate the HR (95% CI) of the association between baseline daily intake of vitamin E and incidence of dementia by 4 vitamin E intake groups. Model 1 was adjusted for age, gender, smoking, drinking, years of education, APOE, physics activity, cognitive activity, BMI and CESD, and model 2 was further adjusted for total energy intake.

The “rate of annual decline of MMSE score,” which indicates the deterioration speed of global cognitive function across the follow-up period, was calculated as the difference of MMSE scores (the baseline MMSE minus the follow-up MMSE) divided by follow-up years. Multivariate linear regression model was used to examine the correlation of the rate of annual decline of MMSE with vitamin E intake, adjusting for age, gender, smoking, drinking, years of education, APOE, physics activity, cognitive activity, BMI, CESD, and total energy intake. Residual plots were used to check the linearity assumption of the linear regression models.

R 4.1.2 and SPSS 25 statistical software were used for data analysis. All *P*-values and 95% CI were two-sided. *P* < 0.05 was considered as the difference or association was statistically significant.

## Results

As shown in Table 1, 1,550 participants were followed up for average 5.2 (SD 0.9) years (8111.3 person-years). The average age of the participants was 71.05 years, and 53.8% of the participants were female. The average daily intake of vitamin E of the participants was 17.54 mg/d, which was closed to the dietary reference intakes (DRIs) for Chinese population (31). At baseline, age, gender, cigarette smoking, years of education, physical activity, cognitive activity, BMI, and CESD were significantly different in four vitamin E intake groups. During the follow-up, 135 (8.7%) participants developed dementia, and the average annual decline rate of MMSE was 0.35 (SD 1.0) in all participants. The rates of incident dementia and the annual decline rates of MMSE were also significantly different among the four groups (*P* < 0.001).

**TABLE 1:** Characteristics of participants by groups of vitamin E intake.

	Total	Low intake (<13.20 mg/d)	Low-medium intake (13.20∼17.54 mg/d)	Medium-high intake (17.54∼23.63 mg/d)	High intake (>23.63 mg/d)	
					
	(*n* = 1550)	(*n* = 387)	(*n* = 388)	(*n* = 388)	(*n* = 387)	P-value*
*Baseline*						
Age, mean (SD)	71.05 (7.1)	73.66 (7.5)	71.11 (6.8)	70.88 (7.0)	68.56 (6.1)	<0.001
Sex, female, n (%)	834 (53.8)	233 (60.2)	218 (51.2)	205 (52.8)	178 (46.0)	0.001
Cigarette smoking, n (%)	164 (10.6)	36 (9.3)	31 (8.0)	45 (11.6)	52 (13.5)	0.065
Alcohol drinking, n (%)	143 (9.2)	30 (7.8)	38 (9.9)	36 (9.3)	39 (10.1)	0.697
Education year, mean (SD)	11.94 (4.0)	11.00 (4.5)	11.94 (4.0)	12.19 (3.9)	12.63 (3.5)	<0.001
APOE ε4+, n (%)	259 (16.7)	62 (16.0)	68 (17.5)	60 (15.5)	69 (17.8)	0.777
Physical activity (METs), mean (SD)	25.73 (24.9)	21.49 (22.5)	25.35 (23.4)	25.29 (26.9)	30.80 (25.9)	<0.001
Cognitive activity, mean (SD)	39.42 (12.0)	34.98 (11.9)	38.98 (11.7)	40.68 (12.0)	43.09 (11.1)	<0.001
BMI, mean (SD)	24.67 (3.5)	24.15 (3.6)	24.51 (3.3)	25.07 (3.5)	24.95 (3.4)	0.001
CESD, mean (SD)	7.88 (7.8)	8.70 (8.2)	7.99 (7.9)	7.51 (7.3)	7.33 (7.7)	0.068
Total energy intake, kcal/d, mean (SD)	1254.93 (685.0)	896.25 (230.8)	1121.20 (261.7)	1275.55 (323.0)	1727.85 (1133.6)	<0.001
Polyunsaturated fatty acid intake, g/d, mean (SD)	5.36 (7.5)	2.91 (1.2)	4.22 (1.5)	5.17 (1.8)	9.15 (14.1)	<0.001
Carotene intake, μg/d, mean (SD)	2973.49 (2206.7)	2035.96 (940.1)	2619.31 (1211.8)	2981.80 (1423.9)	4259.68 (3533.9)	<0.001
Vitamin C intake, mg/d, mean (SD)	94.50 (61.5)	61.83 (26.7)	80.42 (23.8)	93.88 (29.1)	141.98 (97.5)	<0.001
*Follow-up*						
Incident dementia cases, n (%)	135 (8.7)	55 (14.2)	30 (7.7)	33 (8.5)	17 (4.4)	<0.001
Rate of annual MMSE decline, mean (SD)	0.35 (1.0)	0.53 (1.5)	0.31 (1.0)	0.34 (0.8)	0.21 (0.6)	<0.001

*Comparison among the four groups; APOE, apolipoprotein E; BMI, Body mass index; CESD, Center for Epidemiologic Studies Depression Scale.

**FIGURE 1:**
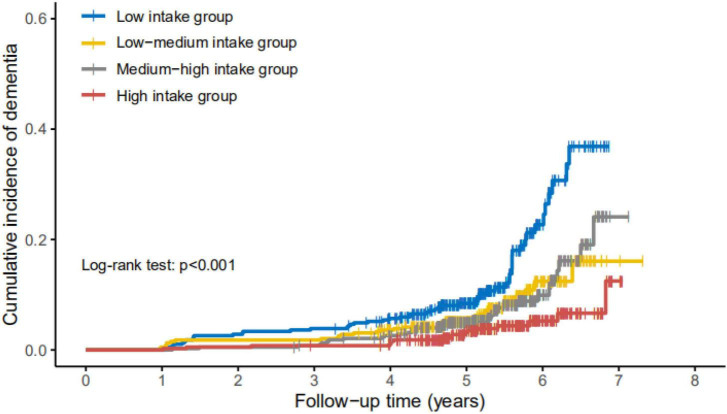
Cumulative incidence of dementia in participants with different levels of daily intake of vitamin E. Cumulative incidence rate of dementia increased most dramatically during the follow-up in participants with vitamin E low intake (<13.20 mg/d, *n* = 387), followed by low-medium intake group (13.20–17.54 mg/d, *n* = 388), medium-high intake group (17.54 and 23.63 mg/d, *n* = 388), and high intake group (>23.63 mg/d, *n* = 387), resulted by Kaplan–Meier curve with log-rank test, *p* = 0.044.

Cumulative incidence rate of dementia increased most dramatically during the follow-up in participants with vitamin E low intake (<13.20 mg/d), followed by low-medium intake, high-medium intake, and high intake groups (log-rank test, *p* = 0.044) (Figure 1).

As shown in Table 2, compared to participants with high vitamin E intake (>23.63 mg/d), the estimated HRs of incident dementia in low intake, low-medium intake, and medium-high intake groups were 2.31 (95% CI 2.20–4.13), 1.80 (95% CI 0.98–3.32), and 1.75 (95% CI 0.95–3.21), respectively, adjusting for age, gender, drinking, smoking, years of education, APOE, physical activity, cognitive activity, BMI, and CESD. Low vitamin E intake group still showed significantly higher risk of incident dementia (HR 2.34, 95% CI 1.20–4.57) after further adjustment of total energy intake.

**TABLE 2:** Association between vitamin E intake level and risk of dementia.

Vitamin E intake level	Person-years	Incidence, per 100 person-years	Model 1*		Model 2**	
				
			HR (95% CI)	*P-value*	HR (95% CI)	*P-value*
Low intake group (<13.20 mg/d)	1955.3	2.8	2.31 (2.20, 4.13)	0.005	2.34 (1.20, 4.57)	0.013
Low-medium intake group (13.20∼17.54 mg/d)	1990.5	1.5	1.80 (0.98, 3.32)	0.059	1.67 (0.86, 3.25)	0.130
Medium-high intake group (17.54∼23.63 mg/d)	2066.7	1.6	1.75 (0.95, 3.21)	0.072	1.84 (0.99, 3.41)	0.054
High intake group (>23.63 mg/d)	2098.8	0.7	1		1	

*Multivariate Cox regression model 1, adjusting for age, gender, smoking, drinking, years of education, APOE, physics activity, cognitive activity, BMI, and CESD. **Multivariate Cox regression model 2, adjusting for total energy intake, age, gender, smoking, drinking, years of education, APOE, physics activity, cognitive activity, BMI, and CESD.

**FIGURE 2:**
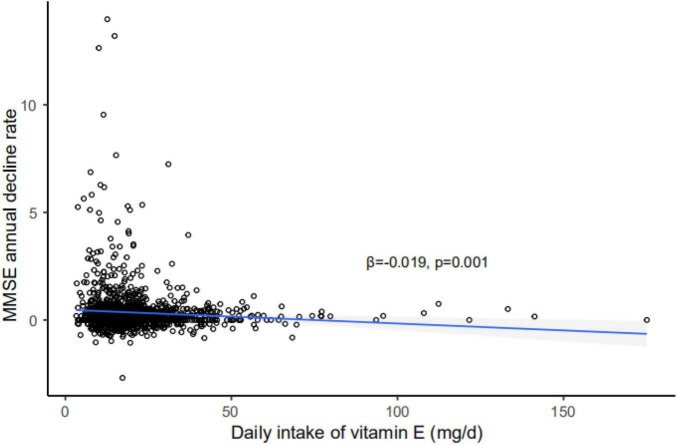
Correlation of annual MMSE decline rate with daily intake of vitamin E. Vitamin E intake was negatively related to the rate of annual decline of MMSE, after adjusted for age, gender, smoking, drinking, years of education, APOE, physical activity, cognitive activity, BMI, CESD, and total energy intake. Shadow represents 95% confidence interval (CI); solid line represents regression line; MMSE, Mini-Mental State Examination; APOE, apolipoprotein E.

Vitamin E intake was negatively correlated to the deterioration speed of global cognition represented as the rate of annual decline of MMSE, after adjusted for age, gender, smoking, drinking, years of education, APOE, physics activity, cognitive activity, BMI, CESD, and total energy intake (β = 0.019, *p* = 0.001) (Figure 2).

## Discussion

Our study found a negative association between dietary vitamin E intake and risk of incident all-cause dementia, and it was also negatively correlated to the rate of annual decline of MMSE after controlling for confounders. To our knowledge, this is the first prospective community-based study with the evidence of the association between dietary vitamin E intake and risk of cognitive decline among Chinese older adults. The strengths of our study were the long-term prospective study design, and the consensus diagnosis based on a comprehensive evaluation including neurological examination, functional evaluation, and neuropsychological assessment.

Our findings are consistent with that from the Memory and Aging Project, in which a cohort of 960 participants aged 58–99 years completed a FFQ and ≥2 cognitive assessments over a mean 4.7 years. It was found that consumption of approximately 1 serving per day ofα-tocopherol (from 4.3 to 8.1 mg/d) may slow cognitive decline with aging (β = 0.03, *p* = 0.020) (32). Several previous studies reported the association between blood concentration of vitamin E and cognitive impairment. A meta analysis (33) including 904 AD patients and 1,153 controls found that AD patients had a lower concentration of serum vitamin E than that of healthy controls. The Cardiovascular Risk Factors, Aging, and Dementia (CAIDE) in Finland (34) followed 140 non-cognitively impaired older participants for 8 years, and showed that lower risk of cognitive impairment in those with higher levels of γ-tocopherol, β-tocotrienol, and total tocotrienols. The InCHIANTI study (35) examined a cohort of 1,033 participants aged at least 65 years, and found that participants with plasma vitamin E levels in the bottom tertile had a significantly higher probability of being demented [odds ratio (OR) 2.6, 95% CI 1.0–7.1], and also of suffering from cognitive impairment (OR 2.2, 95% CI 1.2–4.2) compared to those in the highest vitamin E tertile.

The effect of vitamin E supplements was also reported previously. The Canadian Study (36) of Health and Aging, in which the data set included 5,269 individuals indicated that, compared with those not taking vitamin supplements, the adjusted hazard ratios of cognitive impairment no dementia, AD, and all-cause dementia were, 0.77 (95% CI 0.60–0.98), 0.60 (95% CI 0.42–0.86), and 0.62 (95% CI 0.46–0.83), respectively, for those taking vitamin E and/or C supplements. In the Honolulu-Asia Aging Study (37) which included 3,385 men aged 71–93 years, a significant protective effect was found for vascular dementia who had reported taking both vitamin E and C supplements in 1988 (OR 0.12; 95% CI 0.02–0.88). They were also protected against mixed/other dementia (OR 0.31; 95% CI 0.11–0.89). Among those without dementia, use of either vitamin E or C supplements alone in 1988 was associated significantly with better cognitive test performance 3–5 years later (OR 1.25; 95% CI 1.04–1.50). However, the Prevention of Alzheimer’s Disease by Vitamin E and Selenium (PREADViSE) trial (38) (HR 0.88, 95% CI 0.64–1.20) and the Adult Changes in Thought study (HR 0.93, 95% CI 0.72–1.20) (39) did not find the significant protective effect of vitamin E supplement for dementia.

In our study, we chose the dietary as the main approach to calculate the amount of daily vitamin E intake. Some studies believe that vitamin E does not play a significant role in the prevention and treatment of AD (12, 13). That may be due to the bioavailability of vitamin E, which is complex, can be influenced by some important factors, such as the intake of competing nutrients, intestinal differences in absorption, age, gender, smoking, obesity, and genetic polymorphisms (40).

Vitamin E has a strong anti-oxidant function. Each of the eight tocopherols and tocopherol homologs is considered to be a free radical scavenger (41). The anti-oxidant function is due to the inhibition of free radicals by hydroxyl groups in the aromatic ring of tocopherol through hydrogen atom donors (42). The anti-oxidant function of vitamin E can protect the cell membrane from oxidative damage when it is rich in highly unsaturated fatty acids, so it plays a key role in protecting the cell membrane (43). In addition to anti-oxidant effects, vitamin E also has the characteristics of affecting gene expression, potential pathological effects of AD, and other neuroprotective, anti-inflammatory, and cholesterol lowering (44). Several studies have shown that Vitamin E supplementation has beneficial effects on various markers of human inflammatory stress, cellular signal, and immune function, and has an impact on related pathology (45, 46). Studies on mouse AD models have identified the association between Vitamin E deficiency and increased expression of genes related to AD progression, including apoptosis, neurotransmission and αβ Genes related to metabolic regulation (47).

There are several limitations in this study. Firstly, we studied the association between vitamin E daily intake level and risk of “all-cause dementia,” not specific types of dementia because further CT/MRI/PET scans and cerebrospinal fluid biomarkers examinations could not be conducted according to the limited funding. It may restrain the significance of the finding in the prevention of specific types of dementia. Secondly, during the follow-up period, the environment of the participants and the occurrence of other diseases may have an impact on the association. Thirdly, we only collected the FFQ at the baseline and used the baseline dietary intake data as the exposure because we think the dietary habits of older participants were relatively stable across the follow-up time. However, the potential change in dietary habits may exist and should be considered during the follow-up. Fourthly, although the multivariate analysis was used to adjust factors such as age, gender, smoking, drinking, years of education, APOE, physics activity, cognitive activity, BMI, and CESD, the impact of other confounders on the incidence of dementia cannot be completely excluded. Other confounders like anti-oxidant nutrients and anti-inflammation nutrient cannot be included in the analysis model because of the limited sample size. However, we analyzed the association between carotene/polyunsaturated fatty acid/vitamin C and incident dementia adjusting for age, sex, education years, and APOE, and found that higher vitamin C intake was associated with lower dementia risk. These preliminary results suggest that anti-oxidant nutrients may be one of the important related factors that should be further considered. We did not control for the intake of compounds that could potentially interfere with the bioavailability, absorption and/or metabolic turnover of vitamin E which may also affect the estimation of the associations. Fifthly, although the current study excluded individuals with dementia in order to obtain the dietary data as accurately as possible, the recall bias might still have occurred among those with mild cognitive impairment (approximately 20%). However, this may be less vulnerable to influence the results. Finally, there is a potential representative bias of our study samples because we selected a cohort of older adults with an average age of 70 years old and living in downtown Shanghai, the largest and most developed city in China. Therefore, our results may not be generalized to other populations.

In conclusion, our study found that vitamin E intake was negatively correlated with the risk of incident dementia. Our results suggest that, in the older population, an appropriate high amount of vitamin E intake from the diet might be helpful to prevent future cognitive decline. Prospective studies with larger sample size, longer follow-up, multi-dimensional measurement of vitamin E concentration in human body, i.e., intake level and blood concentration, should be carried out to further understand the impact of vitamin E on the risk of cognitive impairment in the older adults.

## Data availability statement

The data in the current study are available from the corresponding author on reasonable request and with permission of Huashan Hospital. Requests to access the datasets should be directed to DD, dding99@yahoo.com.

## Ethics statement

The studies involving human participants were reviewed and approved by the Medical Ethics Committee of Huashan Hospital at Fudan University (No. 2009-195). The patients/participants provided their written informed consent to participate in this study.

## Author contributions

JL and DD: study conception and design. QZ, ZX, and DD: clinical diagnosis and interpretation of the data. SL, JL, WW, XZ, SD, YW, and DD: acquisition, analysis, or interpretation of data. SL, XL, and JL: statistical analysis. SL, JL, and DD: manuscript drafting. QZ and DD: critical revision and commentary on manuscript. All authors read and approved the final manuscript.
